# Frontiers of cancer care in Asia-Pacific region: cancer care in Australia

**DOI:** 10.2349/biij.4.3.e30

**Published:** 2008-07-01

**Authors:** ES Koh, VT Do, MB Barton

**Affiliations:** 1 Collaboration for Cancer Outcomes Research and Evaluation (CCORE) University of New South Wales, Liverpool Hospital, Liverpool, New South Wales, Australia; 2 Department of Radiation Oncology, Westmead hospital, Wentworthville, New South Wales, Australia

**Keywords:** Cancer care Australia, health inequalities, cancer outcomes

## Abstract

Cancer has a significant impact on the Australian community. One in three men and one in four women will develop cancer by the age of 75. The estimated annual health expenditure due to cancer in 2000-1 in Australia was $2.7 billion, representing 5.5% of the country’s total healthcare expenditure. An historical overview of the national cancer control strategies in Australia is provided. In males, the five most common cancers in order of decreasing incidence are: prostate cancer, colorectal cancer, lung cancer, melanoma and lymphoma, while for Australian women, breast cancer is the most common cancer. Key epidemiologic information about these common cancers, current management issues and comprehensive national clinical practice guidelines (where available) are highlighted. Aspects of skin cancer, a particularly common cancer in the Australian environment – with a focus on melanoma – are also included.

Cancer outcomes in Australia, measured by selected outcomes, are among the best in the world. However, there is still evidence of health inequalities, especially among patients residing in regional and remote areas, the indigenous population and people from lower socio-economic classes. Limitations of current cancer care practices in Australia, including provision of oncology services, resources and other access issues, as well as suggested improvements for future cancer care, are summarised. Ongoing implementation of national and state cancer control plans and evaluation of their effectiveness will be needed to pursue the goal of optimal cancer care in Australia.

## OVERVIEW OF CANCERS AND CANCER STATISTICS IN AUSTRALIA

Cancer has a significant impact on the Australian community. One in three men and one in four women will develop cancer by the age of 75. In 2004, there were over 98,000 new cases of cancer (excluding non-melanomatous skin cancer [NMSC]) and over 36,000 deaths attributable to cancer in Australia, in a population of just over 20 million people. This represented a 4.7% increase from cases diagnosed in 2003 and a 25.2% increase from 1994 [1]. Both the number of new cases is increasing, as is the number of people living with the diagnosis of cancer [2]. Between 1990 and 2000, there was a 36% increase in new cancer cases, but only a 12% population growth. The annual age-standardised incidence rate is 338 per 100,000 people, with a 1 in 3 risk of developing a cancer before the age of 75 and 1 in 2 by the age of 85 [1].

Cancer is the leading cause of death and the leading cause of loss of Disability Adjusted Life Years. Over 60% of people diagnosed with cancer survive more than five years in Australia, a figure that is second only to the USA. It is estimated that over 247,000 people are living in the Australian community with a cancer diagnosis up to five years earlier (excluding NMSC) [1,3]. The estimated annual health expenditure due to cancer in 2000-1 in Australia was $2.7 billion [4], representing 5.5% of the total healthcare expenditure.

## CANCER CONTROL AND HEALTHCARE POLICY IN AUSTRALIA

Australia has a complex healthcare system. The Australian (Federal) Government subsidises out-of-hospital medical services through its Medicare system [5] created as a universal access healthcare system for Australian citizens. The Federal Government also has responsibility for the breast and cervical cancer screening programs, the Pharmaceutical Benefits Scheme (PBS) [6], and other areas of service provision including radiotherapy services and aged care. Hospital care and some community services are the responsibility of State and Territory governments and these are publicly funded, with private services supported by health insurance plans. Cancer care and cancer control have a large component of non-government and non-profit (voluntary) support, the latter notably including Cancer Councils, based in each State and Territory with a national office, and The Cancer Council Australia.

## NATIONAL CANCER CONTROL STRATEGIES IN AUSTRALIA – A HISTORICAL OVERVIEW

In 1994 the publication ‘*Better Health for Australians*’ [7] was released. Cancer was one of the initial health focus areas selected in recognition of its effects on the Australian population and the potential to reduce morbidity and improve quality of life by improving cancer control. Seven priority cancers were selected: breast, cervical, lung, colorectal, melanoma, non-melanomatous skin cancer and prostate cancer. The Australian Cancer Society (now the Cancer Council Australia) conducted a national series of expert workshops and from this produced the *‘National Cancer Prevention Policy for Australia’* [8-10]. Prevention strategies covered tobacco, ultraviolet (UV) radiation, diet, physical activity, overweight and obesity, and alcohol-related issues. This policy [9] also included goals and strategies related to screening for breast, cervical and colorectal cancer. Consensus-based proposed actions were developed subsequently and published in *‘Cancer Control towards 2002’* [9,11].

In 2003, the Optimising Cancer Care in Australia (OCCA) report [2] compiled with key organisations and individuals in the field of cancer care, aimed to identify systematic problems, barriers and failings of the current system of cancer care in Australia. The OCCA report helped stimulate major action in cancer control in Australia at both national and state levels, and is one of the key documents on which the National Service Improvement Framework (NSIF) for Cancer [2,12] is based. The eight priority actions include establishing:

- Integrated and networked cancer services;
- Accreditation for cancer services and credentialing of practitioners;
- Funding structures to support multi-disciplinary care;
- Approaches to monitor cancer control;
- Provision of consumer information about cancer risks, prevention, early detection, diagnosis and treatment, and supportive care;
- Support for primary care providers to provide appropriate assessment of risk;
- Implementation and evaluation of culturally appropriate programs to improve cancer control; and
- Review of gaps in research and opportunities at least every three years.
- The strengths, weaknesses and future directions of these initiatives will be discussed in a later section.

### Common cancers in Australia

In males, the five most common cancers in order of decreasing incidence are:

1. Prostate cancer
2. Colorectal cancer
3. Lung cancer
4. Melanoma
5. Lymphoma

Tables 1 and 2 outline the incidence and mortality figures for the five most common cancers in Australian males over the period 1996-2004 [1].

**Table 1:** The five most common cancers in Australian males over the period 1996-2004 [4] (Rates are age-standardised to the Australian population and expressed per 100,000 population).

**Cancer site/type**	**1996**	**1997**	**1998**	**1999**	**2000**	**2001**	**2002**	**2003**	**2004**
Prostate	137.6	129.8	128.1	129.5	128.2	130.3	134.4	146.8	163.4
Colorectal	78.3	77.2	74.8	75.2	79.6	78.4	75.7	73.8	75.1
Lung, bronchus & trachea	69.8	69.3	67.5	65.6	63.4	62.1	60.7	58.6	61.6
Melanoma of skin	53.8	56.0	52.3	54.2	54.7	55.5	59.9	58.1	56.6
Lymphoma	23.9	23.4	22.9	23.6	24.1	23.8	24.6	24.4	24.3
All cancers	563.0	553.2	544.0	545.4	544.8	548.7	553.6	556.7	573.4

Notes: (a) Non-melanoma skin cancer (NMSC, ICD-10 code C44), known to be the most common cancer type, is excluded from this list because basal cell carcinoma and squamous cell carcinoma, the two most common types of NMSC, are not notifiable cancers. (b) Rates are age-standardised to the Australian population at 30 June 2001 and expressed per 100,000 population. (c) Source of data: National Cancer Statistics Clearing House, AIHW.

**Table 2:** Mortality figures from 2004 for Australian males for the five most common cancers (Rates are age-standardised to the Australian population and expressed per 100,000 population) [4]

	**New cases**					**Deaths**			
**Cancer site/type**	**Number**	**% of total**	**Rate**	**Risk**		**Number**	**% of total**	**Rate**	**PYLL**
Prostate	15,759	28.7	163.4	1 in 5		2,792	12.9	33.0	6,193
Colorectal	7,160	13.0	75.1	1 in 10		2,196	10.1	23.8	14,483
Lung, bronchus & trachea	5,826	10.6	61.6	1 in 11		4,733	21.8	50.8	28,190
Melanoma of skin	5,503	10.0	56.6	1 in 15		815	3.8	8.7	8,605
Lymphoma	2,352	4.3	24.3	1 in 33		803	3.7	8.8	6,513
All cancers	54,870	100.0	573.4	1 in 2		21,670	100.0	237.5	295,080

Notes: (a) Non-melanoma skin cancer (NMSC, ICD-10 code C44), known to be the most common cancer type, is excluded from this list because basal cell carcinoma and squamous cell carcinoma, the two most common types of NMSC, are not notifiable cancers. However, NMSC is included in the data in the mortality columns. In 2004 there were a total of 360 (including 251 male deaths) from NMSC. (b) Rates are age-standardised to the Australian population at 30 June 2001 and expressed per 100,000 population. (c) Risk in 2004 of being diagnosed with a particular cancer before reaching age 85 years. (d) Potential years of life lost (PYLL) between the ages of 0 and 84 years. (e) Sources of data: National Cancer Statistics Clearing House and National Mortality Database, AIHW.

In females, the five most common cancers in order of decreasing incidence are:

1. Breast cancer
2. Colorectal cancer
3. Melanoma
4. Lung cancer
5. Lymphoma

Tables 3 and 4 outline the incidence and mortality figures for the five most common cancers in Australian females over the period 1996-2004 [1].

**Table 3:** The five most common cancers in Australian females over the period 1996-2004. (Rates are age-standardised to the Australian population and expressed per 100,000 population) [4]

**Cancer site/type**	**1996**	**1997**	**1998**	**1999**	**2000**	**2001**	**2002**	**2003**	**2004**
Breast	109.1	111.4	114.6	111.2	115.6	117.2	117.2	112.2	112.8
Colorectal	52.3	52.5	52.0	53.8	52.7	54.5	51.8	51.5	51.5
Melanoma of skin	38.1	40.3	37.1	37.6	38.4	38.4	40.8	38.0	39.4
Lung, bronchus & trachea	26.4	27.0	26.3	26.2	27.9	28.0	28.9	27.7	29.3
Lymphoma	16.9	17.4	17.1	17.2	17.7	17.0	17.9	17.1	17.5
All cancers	385.9	390.2	392.0	388.0	394.5	396.8	402.7	389.9	395.4

Notes: (a) Non-melanoma skin cancer (NMSC, ICD-10 code C44), known to be the most common cancer type, is excluded from this list because basal cell carcinoma and squamous cell carcinoma, the two most common types of NMSC, are not notifiable cancers. (b) Rates are age-standardised to the Australian population at 30 June 2001 and expressed per 100,000 population. (c) Source of data: National Cancer Statistics Clearing House, AIHW.

**Table 4:** Mortality figures from 2004 for Australian females for the five most common cancers. (Rates are age-standardised to the Australian population and expressed per 100,000 population) [4]

	**New cases**					**Deaths**			
**Cancer site/type**	**Number**	**% of total**	**Rate**	**Risk**		**Number**	**% of total**	**Rate**	**PYLL**
Breast	12,126	27.9	112.8	1 in 9		2,664	15.8	23.8	48,910
Colorectal	5,817	13.4	51.5	1 in 14		1,872	11.1	16.0	21,798
Melanoma of skin	4,219	9.7	39.4	1 in 24		385	2.3	3.4	6,790
Lung, bronchus & trachea	3,270	7.5	29.3	1 in 24		2,526	15.0	22.3	34,770
Lymphoma	1,920	4.4	17.5	1 in 46		736	4.4	6.3	8,725
All cancers	43,466	100.0	395.4	1 in 3		16,819	100.0	145.8	229,483

Notes: (a) Non-melanoma skin cancer (NMSC, ICD-10 code C44), known to be the most common cancer type, is excluded from this list because basal cell carcinoma and squamous cell carcinoma, the two most common types of NMSC, are not notifiable cancers. However, NMSC is included in the data in the mortality columns. In 2004 there were a total of 360 (including 109 female deaths) from NMSC. (b) Rates are age-standardised to the Australian population at 30 June 2001 and expressed per 100,000 population (c) Risk in 2004 of being diagnosed with a particular cancer before reaching age 85 years. (d) Potential years of life lost (PYLL) between the ages of 0 and 84 years. (e) Sources of data: National Cancer Statistics Clearing House and National Mortality Database, AIHW.

The following section summarises some key epidemiologic information about these common cancers in Australia and highlights current issues and methodologies in management. Where available for relevant cancers, national clinical practice guidelines are referenced.

## SKIN CANCER – A COMMON CANCER IN THE AUSTRALIAN ENVIRONMENT

### Melanoma and non-melanomatous skin cancer

The ultraviolet (UV) light in sunlight damages the DNA in skin, causing skin cells to mutate and contribute to the process of carcinogenesis. Humans have evolved a protective mechanism for filtering out UV light from specialised skin cells (melanocytes), which produce a dark pigment called melanin that absorbs UV light and prevents its damaging effects [13, 14]. The mass migration of peoples in the 19th and 20th centuries redistributed populations with low melanin protection to high UV regions. For example, when fair-skinned Anglo-Celtics and Europeans migrated to hotter climates such as that in Australia, the rates of skin cancer increased [14]. Australians have the highest documented incidence of skin cancer in the world, and skin cancer is the most common form of cancer in Australia, where the lifetime risk of skin cancer is 1 in 2 people [1]. Those persons most at risk are:

- Persons with fair skin and blue eyes (having the least amount of melanin)
- Persons with significant outdoor sun exposure such as farm or construction workers
- Urban indoor workers who spend weekends or holidays (or their childhood and young adulthood) in the sun.

### Melanoma

Australia has the highest incidence and mortality rates for melanoma, as summarised in Table 5 demonstrating international trends [15]. The evidence of a causative link with sunlight exposure is compelling, with severe episodic sunburn in early life correlating best with melanoma risk [15-19]. The Sydney Melanoma Unit (SMU) has been the global pioneer in the diagnosis, staging and management of this malignancy [20-22], especially with regard to multi-disciplinary care [20], and its contribution to the formulation of comprehensive clinical practice guidelines published by the National Health and Medical Research Council (NHMRC) [18]. The SMU has been particularly instrumental in defining the role and technique of sentinel lymph node mapping in melanoma [21,23] and has led international collaborative clinical and translational research trials [24].

**Table 5:** Melanoma statistics in four countries [1,4,5,79,80]

	**Age-standardised incidence (100,000/year)**	**Age-standardised mortality (100,000/year)**	**Lifetime risk (incidence)**	**Incidence trend over 10 years**	**Mortality trend over 10 years**	**Most common cancers (ranking)**
**Australia (2001) [1]**						
Men	41·4 (world)	5·1 (world)	1 in 25	22% increase	2% increase (1991–2001)	4th
Women	31·1 (world)	2·6 (world)	1 in 35	12% increase	0% increase (1991–2001)	3rd
**USA (2001) [4,5]**						
Men	21·4 (world)	3·9 (world)	1 in 53	31% increase	0% increase (1991–2001)	5th
Women	13·8 (world)	1·8 (world)	1 in 78	25% increase	1% decrease (1991–2001)	7th
**The Netherlands (1998) [79]**						
Men	11·5 (Europe)	3·1 (Europe)	..	21% increase	24% increase (1989–98)	..
Women	14·8 (Europe)	2·1 (Europe)	..	11% increase	5% increase (1989–98)	..
**UK (2000) [80]**						
Men	9·7 (world)	2·7 (world)	1 in 147	59% increase	20% increase (1991–2001)	12th
Women	11·2 (world)	1·9 (world)	1 in 117	41% increase	3% increase (1991–2001)	7th

### Lung cancer

Although lung cancer is only the third highest cancer in terms of incidence, it is the leading cause of cancer death in males and females, as well as the top cause of ‘Person Years of Life Lost’ due to cancer in Australia. More than 8,000 Australians are diagnosed with lung cancer each year, with about 7,000 deaths secondary to the disease each year. One in 30 Australians will develop lung cancer by age 75 [1]. Relative survival following a diagnosis of lung cancer in the Australian states during the 1980s and 1990s (for which data is available) varied in the ranges of 10.1-11.1% in males and 12.3%-13.7% in females at five years. More recent Australian data for the period 1992-97 show a five-year relative survival of 11% for males and 14% for females. Comparative international trends in lung cancer are outlined in Table 6. Lung cancer incidence is decreasing in males but increasing in females, with cigarette smoking the major cause in up to 90% of cases. In 2003, there were an estimated 10,378 new cases of all cancers and 7,727 deaths from all cancers in Australia attributed to smoking (see below) [1]. Other causes of lung cancer include environmental (passive) smoking (although the risk is less than active smoking), and occupational exposure to asbestos.

**Table 6:** Five-year relative survival from lung cancer – international comparisons [3, 48, 81-84].

			**Years after Diagnosis**				
**Males**	**Time period**	**Age Group**	**1 yr(%)**	**2 yr(%)**	**3 yr(%)**	**4 yr(%)**	**5 yr(%)**
Australia	1992-97	All	34.6	-	-	-	11.0
New South Wales	1980-94	15-89	34.0	18.0	13.4	11.2	10.1
Europe	1985-89	15+	32.0	-	12.0	1.0	10.0
United States (SEER)	1991	All	38.9	21.9	17.0	14.0	12.4
**Females**	**Time period**	**Age Group**	**1 yr**	**2 yr**	**3 yr**	**4 yr**	**5 yr**
Australia	1992-97	All	37.6	-	-	-	14.0
New South Wales	1980-94	15-89	37.3	20.8	15.9	13.5	12.3
Europe	1985-89	15+	29.0	-	13.0	-	11.0
United States (SEER)	1991	All	44.9	27.0	21.4	18.8	16.4

Notes: (a) When comparing survival and mortality data it should be noted that the denominator for survival is the population of patients with disease, whereas the denominator for the mortality rate from lung cancer is the whole population. Thus, the mortality rate may be low is there are a small number of cases with the disease, whereas poor survival results from patients with the disease dying relatively quickly.

#### Management issues in lung cancer

In 2004 the NHMRC published clinical practice guidelines for the prevention, diagnosis and management of lung cancer [25]. The primary reason for the publication of these guidelines was to assist in educating and improving the practice and quality of care provided by practitioners who manage lung cancer patients in Australia. The overall aim of the guidelines was thus to improve consistency of care and patient outcomes. These guidelines underscore the value and importance of multi-disciplinary care, as have publications from other Australian centres [26,27]. To date, no national screening program for lung cancer exists in Australia, based on lack of high-level evidence to support its implementation and use [25]. The NHMRC guidelines contain comprehensive evidence-based recommendations regarding the management of both non-small cell lung cancer and small cell lung cancer by stage [25]. Despite the publication and dissemination of these guidelines, however, there is ongoing evidence of practice variation across Australia. For example, in a survey of 24 radiotherapy departments across the country, there was considerable variation in radiotherapy prescription doses for both radical and palliative treatments, immobilisation techniques, and CT planning-based protocols [28]. Data such as this demonstrate the ongoing need for continued assessment of guideline implementation and also updates as new evidence becomes available.

#### Smoking

Cigarette smoking remains the largest single preventable cause of death and disease including lung cancer. Consequently, tobacco control measures, including taxation and price policies, advertising restrictions, public information, health promotion and smoking cessation support, are pivotal in reducing the burden of disease from smoking. In 2004 the Ministerial Council on Drug Strategy [29] endorsed an action plan under the National Drug Strategic Framework, *The National Tobacco Strategy (NTS), 2004-2009* [30]. The Australian government reiterated its determination to reduce tobacco use by ratifying the World Health Organization’s Framework Convention on Tobacco Control [31].

### Colorectal cancer

Colorectal cancer (CRC) is the second most common cause of death from cancer, after lung cancer. There are about 11,300 new cases and 4,600 deaths from bowel cancer each year in Australia. The lifetime risk of developing bowel cancer is approximately 1 in 18 Australian men, and 1 in 26 women [1]. Risk factors for the development of CRC include increasing age, low fibre diet, history of polyps and /or colitis, and family history of colorectal cancer.

Population-based bowel screening was tested in a national Pilot Program from November 2002 to June 2004. The National Bowel Cancer Screening Program [32] commenced a phased implementation in August 2006. Initially, screening will be offered to those involved in the Pilot Program and Australians turning 55 or 65 years of age between May 1, 2006 and June 30, 2008. Eligible participants will receive an invitation to complete a faecal occult blood test (FOBT). Those returning a positive FOBT result will be advised to discuss it with their family practitioner, who will generally refer them for further investigations, which will usually be a colonoscopy.

#### Management issues in colorectal cancer

In 1999, NHMRC released the ‘*Guidelines for the Prevention, Early Detection and Management of Colorectal Cancer’* [33], with the second edition released in 2005 [34]. The guidelines are intended to provide a resource for all medical practitioners and health workers who require sound information directed toward the management of patients with colorectal cancer. These guidelines are wide-ranging in scope and provide information which covers prevention and screening, diagnosis and psychosocial matters, as well as the clinical aspects of surgery, radiotherapy and chemotherapy. These guidelines have been well adopted, used with demonstrated concordance and implemented into clinical practice [35]. Another significant change in the management paradigm in CRC has been the patterns of follow-up care. In the past, intensive follow-up after treatment was not as strongly promoted. However, it has now been shown that intensive follow-up leads to earlier detection of recurrence. A Cochrane Review demonstrated that it also improves survival [36].

### Breast cancer

Breast cancer is the highest cause of cancer-related deaths among Australian women. The age-standardised incidence of breast cancer in females has increased from 80 per 100,000 population in 1983 to 117 per 100,000 population in 2002. It is projected that there will be 13,261 new cases in 2006 and 14,800 in 2011 in Australia. The lifetime risk of developing breast cancer is approximately 1 in 11 women in Australia. In 2002 there were approximately 114,000 women alive with a past diagnosis of breast cancer [37]. There are various risk factors related to the development of this malignancy, including increasing age, hormonal factors, nulliparity, exogenous oestrogen, a positive family history in first-degree relatives, and previous history of breast cancer or benign breast disease [38].

#### Management issues in breast cancer

The National Breast and Ovarian Cancer Centre (NBOCC) [39] is Australia’s main body for breast and ovarian cancer control, originally established in 1995 by the Australian government. The NBOCC has produced a spectrum of publications and resources including comprehensive evidence-based clinical practice guidelines relating to all aspects of breast cancer and its management. These aspects include screening and early detection, the management of early, advanced, and metastatic breast cancer [38], radiotherapy and breast cancer, the psychosocial care of adults with cancer, and multi-disciplinary cancer care [39].

The BreastScreen Australia public mammography screening program [40] commenced in most Australian states in 1991. Increased cancer incidence rates were seen in the targeted age groups between 50 and 69, and were greatest in the 60-64 age group. The incidence rates increased from 216 per 100,000 population in 1992 to 334 per 00,000 population in 2002. Table 7 demonstrates a global ranking of incidence and mortality for breast cancer in females with data from selected countries [3, 41].

**Table 7:** Global ranking of incidence and mortality for breast cancer in females, selected countries, 2002 GLOBOCAN. (Rates expressed per 100,000 populations and age-standardised to the year 2002 Standard Population of country and to World Standard Population (ASR (W)) [3].

	**Incidence**			**Mortality**		
**Population (Female)**	**Numbers**	**Crude**	**ASR (W)**	**Numbers**	**Crude**	**ASR (W)**
World	1,151,298	37.4	37.4	410,712	13.3	13.2
More Developed Countries	636,128	103.7	67.8	189,765	30.9	18.1
Less Developed Countries	514,072	20.9	23.8	220,648	9.0	10.3
Australia	11,176	114.1	83.2	2,667	27.2	18.4
Canada	19,540	124.0	84.3	5,305	33.7	21.1
New Zealand	2,330	120.0	91.9	670	34.5	24.5
United Kingdom	40,298	135.5	87.2	13,303	44.0	24.3
United States of America	209,995	143.8	101.1	42,913	29.4	19.0
Central and Eastern Europe	100,262	63.4	42.6	45,310	26.7	17.9
Northern Europe	62,425	128.8	82.5	19,789	40.8	22.6
South-Eastern Asia	58,495	21.8	25.5	26,818	10.0	11.8
Southern Europe	72,458	97.8	62.4	24,617	33.2	18.1
Western Europe	125,604	134.3	84.5	39,297	42.0	22.3

Notes: 1. Cancer numbers and rates are estimates for the middle of 2002, from the most recent data available, generally 3-5 years earlier. 2. Rates are expressed per 100,000 populations and age-standardised to the year 2002 Standard Population of the corresponding country and to the World Standard Population (ASR (W)). 3. The Age-Standardized Rate (ASR, world standard) is calculated using the 5 age-groups 0-14,15-44,45-54,55-64,65+ years.

### Prostate cancer

In Australia, prostate cancer is the second most common type of cancer in men (after skin cancer). Relatively little is known about the aetiology of prostate cancer in some men. It is a disease of older men, being rare under the age of 50. When it does occur in men under 50, it is more likely in a man with a family history of prostate cancer. Australians may be more prone to develop prostate cancer as it has been found to be associated with certain diets – including those high in animal fats, low in plant food, and possibly low in certain elements, antioxidants and vitamins – as well as occupational exposure to certain substances, including cadmium and rubber [42].

While variations on the risk strata exist, for the purpose of this review, the American Urological Association (AUA) scheme is referenced, as follows [43]:

- *Low risk*: PSA≤ 10ng/ml, Gleason score ≤ 6 and clinical stage T1C or T2a
- *Intermediate risk*: PSA> 10ng/ml to 20ng/ml or a Gleason score of 7 or clinical stage T2b
- *High risk*: PSA>20 ng/ml or a Gleason score of 8 to 10 or clinical stage ≥T2c

#### Management issues in prostate cancer

The National Health and Medical Research Council (NHMRC) is Australia's main body for supporting health and medical research, and for developing health advice for the Australian community, health professionals and governments. In 2002, the NHMRC released the “*Clinical practice guideline: Evidence based information and recommendations for the management of localised prostate cancer*” [42]. Publication of these evidence-based guidelines has been particularly important in outlining management options and controversies, when weighing up potential treatment-related benefits with relevant morbidities.

#### Localized low- to intermediate- risk prostate cancer

Options for the management of localised prostate cancer include radical prostatectomy, radiotherapy and active surveillance. Radiotherapy includes external beam and interstitial radiotherapy (brachytherapy) treatments. These interventions are options for the treatment of localised prostate cancer because the data currently available in the literature does not provide sufficient clear-cut evidence to indicate the unquestioned superiority of any one form of treatment [42].

#### Localised high-risk prostate cancer

Although active surveillance, non-nerve sparing prostatectomy, high-dose rate brachytherapy and high-dose external beam radiotherapy remain options for the management of patients with high-risk localised disease, recurrence rates are also high. Based on results of randomised controlled trials, the use of hormone therapy in combination with conventional radiotherapy may prolong survival [43].

#### Hormone-refractory prostate cancer

Although hormonal manipulations, such as luteinising hormone-releasing hormone (LHRH) agonists or castration, are initially effective for 90% of prostate cancer patients, all eventually progress after a median of 18-24 months of treatment to become “androgen-independent” (or hormone-refractory). Upon progression, secondary hormonal manipulations are often employed; however, these treatments are generally less effective, and any anti-cancer effects are usually short-lived. The intravenous chemotherapy agent Docetaxel has been used in the treatment of HRPC and demonstrated a survival benefit in selected patients [44].

## INEQUALITIES IN CANCER SUSCEPTIBILITY AND HEALTH OUTCOMES

Despite the fact that Australia ranks among the countries with the lowest mortality rates overall, there are defined sub-groups of the population with documented inferior outcomes, particularly women residing in more regional and remote geographic areas (see later section on access issues) [45], those of lower socioeconomic status [41] (see Table 8), and those of indigenous backgrounds [46,47].

**Table 8:** Breast cancer in females: age-standardised rate and five-year relative survival proportions by region and socioeconomic status, Queensland, 1996-2002. [45].

**Characteristic**	**Incidence(Average number ofcases per year)**	**ASR(Age-Standardized Rate)**	**Five-yearrelative survival(%)**
**Geographic area**			
Major city	1,087	119.5	86.6
Inner regional	575	120.3	87.0
Outer regional	280	99.9	85.8
Remote	33	89.5	81.9
**Socio-economic status (SES)**			
Affluent	143	129.4	88.1
Middle 80% SES	1,716	115.5	86.5
Disadvantaged	115	106.2	84.7

Source: Geographical differentials in cancer incidence and survival in Queensland, 1996 to 2002 [45]. Notes: (a) ASR is the Age-Standardized Rate (b) Relative survival compares the survival of persons diagnosed with cancer (observed) with that experienced by the same age- and sex-matched population to which they belong (expected). The ratio of observed to expected is used to estimate the proportion of people whose risk of dying has been affected by the disease. This method of analysis does not require knowledge of the cause of death.

### Indigenous Australians

Indigenous Australians who do not live in the cities are particularly disadvantaged in accessing radiotherapy. Their strong links to place, family and culture mean that travelling to urban centres for radiotherapy is a significant problem. The greater differences in death rates, compared to incidence rates, between indigenous and non-indigenous people could reflect a higher proportion among indigenous people of cancers with high case-fatality rates, a generally more advanced stage of cancer at time of diagnosis, or differences in treatment outcomes by stage of cancer at diagnosis [48]. In a study of 815 indigenous and 810 non-indigenous people living in Queensland and diagnosed with cancer from 1997-2002, the likelihood of death from cancer was 30% higher for indigenous cases than for non-indigenous cases after accounting for cancer stage at diagnosis, treatment, and the higher rates of co-morbidities (such as diabetes, chronic renal disease, respiratory disease and acute coronary conditions) existing among indigenous cases [49]. Analyses of cancer and cancer services for Indigenous people in the Northern Territory have highlighted the fact that the absolute differences in survival after diagnosis with cancer are greatest for cancers with the highest survival in non-indigenous people [46]. A 2004 review concluded that ”the experience of indigenous people and cancer provides evidence that the Australian health system is not operating as effectively for indigenous people as for other Australians” and that there was a need for “strengthening primary healthcare services, reducing barriers for access to specialist services and improving collaboration between the two” [46].

### Geographic differences

There is increasing evidence to suggest that more than half a million Australians who live outside state capital cities [50] are at risk of significantly poorer survival outcomes following a cancer diagnosis, than people with similar diagnoses who reside in major metropolitan areas [51]. People with cancer living in remote and rural areas are diagnosed at a later stage than their city counterparts [52], and moreover, are more likely to die from cancers such as lung, cervix, and uterine malignancies the further they are located from city centres [53]. Specific indicators of reduced access to cancer care services in remote and rural areas include poorer state-of-the-art diagnostic tests, staging and treatment of prostate cancer [54], less breast-conserving surgery [55], and lower probability of completing external beam radiotherapy when referred for treatment of rectal cancer [56]. As recently as 2004, there was ongoing published evidence in the Medical Journal Australia demonstrating that people with cancer residing in regional New South Wales were 35% more likely to die within five years of diagnosis than patients residing in urban centres [52]. Mortality rates increased with remoteness to cancer facility.

### Geographic differences and socio-economic status (SES)

In 2006 the AIHW published a comprehensive overview report [41] addressing epidemiologic aspects and clinical outcomes in breast cancer in Australia. The most recent data linking geographic differences and SES come from the state of Queensland (see Table 8). ‘Major city’ and ‘inner regional’ areas had higher five-year survival rates with 86.6% and 87.0% survival respectively, compared with ‘Outer regional’ and ‘Remote’ areas with five-year survival rates of 85.8% and 81.9% survival, respectively, in the 1996-2002 period. Over this same period, ‘Affluent’ areas of Queensland had the highest five-year survival rates with 88.1% compared to ‘Middle 80% SES’ areas with 86.5% survival and the ‘Disadvantaged’ areas with 84.7% five-year relative survival [41].

## ADEQUACY OF FACILITIES AND SERVICES BASED ON THE IDEAL PHILOSOPHY OF CANCER CARE

This section will address cancer services by specialty type – surgical, medical and radiation oncology, and palliative care services.

### Surgical oncology services

Surgical expertise tends to be focused on anatomical sites rather than types of pathology. Consequently, individual surgical specialists treat varying proportions of neoplastic and non-neoplastic conditions. A national report is currently being prepared with respect to surgical oncology services, by linking data from Medicare on MBS claims for surgical procedures identified as being primarily cancer-related and also documenting the geographic distribution of these claims.

### Complexity of a surgical oncology intervention, volume of procedures and outcome

There is increasing evidence that outcome of cancer care, particularly for complex difficult primary surgery, is linked to the volume of interventions (for example, operations) undertaken. In Australia there is, for example, evidence showing volume-outcome benefits for individual colorectal surgeons when undertaking difficult rectal surgery, though this difference is not apparent for less difficult surgery [34].

### Medical oncology services

The estimated number of medical oncologists per new case of cancer in 2007 varied considerably, with most states and territories (except Victoria) experiencing an apparent shortfall. The Australian Medical Workforce Advisory Committee (AMWAC) reported that, in 2001, there were 14 medical oncologists and haematological oncologists per million population in Australia. AMWAC recommended that this number be increased to 16 per million population, but did not identify the ratio for each specialty separately [57].

### Availability of chemotherapy drug agents

Cancer treatment accounts for 6% of the health expenditure. Even drug costs, about which much is heard, are quite modest. The cost of anti-cancer drugs is only 15% of the most expensive drug group (lipid-lowering agents) and 2.7% of the total expenditure on the Pharmaceutical Benefit Scheme (PBS) [6]. Indirect costs, that is, costs other than the healthcare costs, are unmeasured and often ignored. For a devastating disease like cancer, these are generally much greater than the cost of treatment [58].

Chemotherapy is mostly delivered by intravenous administration. It is provided in a variety of hospital, as well as public- and private-sector outpatient settings throughout Australia, including many rural locations. In most settings, chemotherapy regimens are determined by medical oncologists but are administered by nursing staff, under the supervision of either a medical oncologist or an appropriately qualified physician.

In 2006, more than 300,000 Medicare claims were made for chemotherapy administration throughout Australia. The number of claims per new case of cancer varied greatly among the states and territories. The PBS Schedule is part of the wider Pharmaceutical Benefits Scheme [34] managed by the Department of Health and Ageing and administered by Medicare Australia, which provides medicines to be dispensed to patients at a government-subsidised price. To explore usage of chemotherapeutic agents, we examined data on PBS services for two selected high-cost drugs, both relatively new agents approved for defined indications on the basis of evidence from randomised controlled trials [59, 60].

1. Trastuzumab, a monoclonal antibody treatment indicated in the management of HER2-*neu* positive breast cancer;
2. Docetaxel, an agent indicated for the adjuvant treatment of node-positive breast cancer in combination with an anthracycline, for the treatment of locally advanced or metastatic breast cancer as second-line chemotherapy, metastatic hormone-refractory prostate cancer [60], advanced, metastatic ovarian cancer, and second-line therapy in locally advanced or metastatic non-small cell lung cancer.

The Federal Government accepted a recommendation from the Pharmaceutical Benefits Advisory committee (PBAC) to list Trastuzumab on the PBS, as of October 1, 2006, for a maximum period of 12 months. Of the 14,000 women who are diagnosed with breast cancer annually in Australia, around 2100 are expected to be treated with Trastuzumab per year. The calculated costs of the 52 weeks of treatment was estimated to be in the order of $50,000 per eligible patient, with the listing of Trastuzumab anticipated to add $470 million to the PBS expenditure between 2006-7 and 2009-2010 [61] .

Similarly, with respect to Docetaxel, extension of its use in the setting of HRPC was initially rejected by the PBAC in July 2005 because of uncertain and unacceptable cost-effectiveness. In a re-submission in November 2006, its use was approved in HRPC. On updated economic evaluation, its cost was estimated to be in the range of $15,000-$45,000 (intention-to-treat population). The likely number of patients was estimated to be less than 10,000 in Year 4. The financial cost/year to the PBS was estimated to be in the range of $30-60 million in Year 4 [62].

### Radiation oncology services

There is a significant deficit in radiotherapy resources in Australia, both in staffing (radiation oncologists, medical physicists, and radiation therapists) and in equipment. Waiting lists are an obvious outcome measure of the resource shortage, and long waiting time for various cancers have been documented in Australia. Evidence for this exists in clinical settings for some tumour sites, such as post-operative head and neck cancer, small cell lung cancer, high grade cerebral gliomas [63] and cervix cancer (64), where tumour control may be adversely affected [65]. In 2001 and 2002, the Royal Australian and New Zealand College of Radiologists performed audits to specifically address waiting times for radiotherapy in Australia [66]. The results show a steady decline in the number of patients commencing treatment in a timely fashion, with approximately 40% of patients receiving curative treatment, 30% receiving palliative treatment, and 56% receiving emergency treatment starting outside of standard good practice times. The Collaboration for Cancer Outcomes Research and Evaluation (CCORE) has reported an evidence base to support a utilisation rate of 52.2%, resulting in a disparity of approximately 18% between those cancer patients who should – and those who actually do – receive radiation treatment [67]. In 2002, this represented approximately 15,000 patients in Australia who could have potentially benefited from treatment, but were unable to access radiotherapy services.

## LIMITATIONS OF CURRENT CANCER CARE PRACTICE IN AUSTRALIA

Optimizing Cancer Care in Australia is a consultative report prepared in 2003 by the Clinical Oncological Society of Australia, The Cancer Council Australia and the National Cancer Control Initiative, outlining key reforms required to ensure optimal treatment for cancer patients [2]. It identifies three key areas where the “health system” has failed to provide optimal care for cancer patients. These key areas are: models of cancer care, quality of cancer care, and resource issues in cancer care.

### Models of cancer care

#### Traditional versus integrated multidisciplinary care (IMDC)

Medicare and the private health insurance system cater for the traditional model of care where the general practitioner (GP) refers a patient to a specialist (usually a surgeon) who conducts the primary intervention, usually the removal of a tumour. Patients may then see other cancer specialists sequentially for opinions before (but more often after) the primary intervention. This traditional model is criticised for its dependence upon the primary specialist reaching a conclusion that further referral is necessary. They perceive too great a risk of suboptimal therapy unless there is a more formalised method of accessing ‘integrated multidisciplinary care’ (IMDC). Much work has been invested in developing and implementing this concept and model of care. This work has been summarised in reports issued by the National Breast and Ovarian Cancer Centre [39], and includes the management of colorectal cancer [33, 34], breast [38, 39], lung [25, 26] and other malignancies such as melanoma [18].

### Quality of cancer care

#### Improving quality of cancer care

Several major initiatives are underway in Australia which seek to assess mechanisms to improve quality in cancer care. What is striking about these initiatives is that there are relatively so few of them, especially for cancers other than breast cancer. Other drivers for quality could include the Medicare Benefits Schedule (MBS) [68]. The schedule may need to change to promote quality care. Consideration needs to be given to reviewing the MBS items with a view to promoting higher quality cancer care [69].

#### Improving quality through information and research

There are many gaps in our knowledge of the cancer care process, such as how advanced the disease is at diagnosis, how people are treated, as well as how treatment and potential complications affect their quality of life. Researching these issues is vital to our ability to provide optimal care. Funding for this type of research is relatively limited, but it is needed to engage in a dialogue between cancer clinicians and healthcare policy makers to help ensure services are both meeting the needs and are cost-effective.

#### Quality of healthcare providers

- Lack of standards in training curricula and a focus on process instead of the education system’s capacity to deliver positive healthcare outcomes.
- No credentialing of individual practitioners nor accreditation of healthcare services.
- Poor working conditions, due in part to patient through-puts based on an individual clinician’s willingness to meet demand rather than national standards determined by population need and each hospital’s demonstrated capacity.
- Inadequate arrangement for clinical training.
- Unstructured career paths and lack of diversity in healthcare professional roles.

### Resource issues in cancer care

#### Work force (shortages, roles, training, communication skills)

The cancer care workforce faces shortages in almost every category. There are shortages of specialised cancer nurses, radiation therapists, medical physicists, pharmacists and all cancer specialist clinicians (surgical oncologists, medical oncologists and radiation oncologists). The shortages show themselves most acutely in the regional areas outside major capital cities. General practice also requires development, as cancer is increasingly being treated in the community. Communication is widely recognised as problematic, especially as Australia has a culturally and linguistically diverse background.

#### Access issues

An unprecedented increase in cancer incidence and prevalence has lead to marked disparities in cancer mortality and morbidity across population groups (eg poorer outcomes for indigenous, rural and remote communities) as discussed earlier. This is acutely shown in access to cancer care in rural and regional centres.

A number of key access issues affect the quality of cancer care.

#### (1) Access to radiotherapy units

Radiation oncology is in a period of unprecedented change due to increases in the complexity of treatment and improvements in the diagnosis and staging of cancer. Such changes include the expanding use of Magnetic Resonance Imaging (MRI), Ultrasonography, Positron Emission Tomography (PET) and Single Photon Emission Computed Tomography (SPECT) scanning, and the need for image fusion, multi-leaf collimators and conformal therapy, 3D-planning, and intensity modulated radiotherapy (IMRT) – all of which will increase the demands for improved patient immobilisation [70]. The delay in funding for new technologies, due to government bodies waiting for evidence-based data to show an improved outcome, thus creates significant delays in implementation especially for radiotherapy. In the context of radiation oncology practice, creditable results may require many years of follow-up.

The Royal Australian and New Zealand College of Radiologists (RANZCR) routinely surveys waiting times. In May 2001, 44% of patients surveyed started treatment within the optimum waiting time, 30% were within acceptable times, and 26% were outside the acceptable range [66]. Australian Council on Healthcare Standards data show that the proportion waiting more than three weeks for radiotherapy has doubled between 1999 and 2001 to 20% of people. The National Strategic Plan for Radiation Oncology (Australia) identified a lack of access to radiotherapy treatment centres, creating considerably real and present hardship for many patients [71].

#### (2) Access to pharmaceuticals (drugs)

As outlined earlier, cancer drugs that are difficult to access fall into three categories: new drugs that are not yet approved either by Pharmaceutical Benefits Advisory Committee or the Therapeutic Goods Administration (TGA) for that indication; older drugs that have not been approved for the use that they are being applied to; or older drugs that are no longer on the Australian Register of Therapeutic Goods.

#### (3) Access to services including palliative care

Travelling to a treatment centre is a serious barrier to access. Substantial cost is incurred in accessing treatment and some people cannot afford it, especially for prolonged periods of radiotherapy. Greater access to home care, better access to psychological support, and support for carers and families can all decrease the impact of cancer. Although overall access to palliative care in Australia is adequate, referral to palliative care units is often too late or only during a crisis, and one third of the potential population is never referred [2]. One common problem is late referral, which is closely related to the failure to involve palliative care practitioners in care-planning at an earlier stage. Part of the solution is to educate specialists, GPs and the community about palliative care and to change its image. This is particularly pertinent for special groups such as Australian Aboriginals and those from non-English speaking backgrounds, ensuring that they know about the services and how to access it.

## POTENTIAL SOLUTIONS AND FUTURE IMPROVEMENTS IN CANCER CARE AND SERVICE DELIVERY

In the 2004-5 Federal budget, almost $190 million in funding was pledged to be made available over a five-year period until 2008-9 for the ‘*Strengthening Cancer Care’* initiative. Most of the states and territories in Australia have now developed their own local cancer control plans. In particular, the two most populous states, Victoria and New South Wales, have produced a ‘*Cancer Services Framework for Victoria’* [72] and the NSW Cancer Plan 2004-2006, respectively [73]. This plan outlined 33 specific goals in the 10 strategic areas, covering:

- Coordination of cancer control
- Cancer prevention and early detection
- Cancer service provision – the patient’s journey
- Special issues in cancer care
- Cancer information
- Cancer education
- Cancer workforce
- Cancer research
- Cancer fundraising
- Quality, evaluation and accreditation

The *strengths* of these successive comprehensive national and state-based initiatives and reports, have been to identify priority cancers and strategies that have the potential to improve cancer outcomes, decrease morbidity and mortality and address inequalities in cancer care [2]. However there are several major areas of *weaknesses* in these initiatives. These relate to the relative lack of implementation plans, the absence of dedicated funding for implementation, too few economic analyses supporting priority actions and strategies, and little or no evaluation of the uptake or impact of cancer control plans [74]. These challenges are magnified by the fact that those responsible for producing these plans are not always the same groups who have the authority to execute and implement them, and who may have differing priorities. For example, the Cancer Institute NSW has control over a very small part of NSW’s health expenditure on cancer. This is in contrast, notably, with the British National Health Service, which has both the authority to both produce strategic plans and implement them [74]. Other weaknesses include the lack of well-structured implementation strategies and a clear assignment of responsibility and accountability mechanisms.

As outlined in the COSA report [75], cancer care in Australia is thus compounded by associated population pressures, and faces the following demographic challenges and systemic problems:

- Fragmentation of the system across multiple tiers, compromising efficiency of training, planning, recruitment and retention processes.
- Established priorities based on requirements of disparate organisations rather than on Australia’s national healthcare needs as shown by epidemiological data.
- Bureaucratisation and politicisation of government-funded health services.
- No national framework to facilitate staff movement or re-entry across the system.
- No infrastructure for ushering in, and adapting, to rapid technological change.
- No national, independent approach to data collection and use.

### Optimising Cancer Care

The Optimising Cancer Care in Australia (OCCA) report proposed 12 key recommendations and 19 actions items. These related to the key areas of change: models of cancer care, quality of cancer care, resources issues in care and improving the delivery of cancer care [2]. The recommendations emphasised integrated multidisciplinary care, care throughout the cancer journey, including palliative and supportive care, and improved consumer access to information. There were also recommendations for the development of an accreditation system for cancer services, improved access to clinical trials, psycho-oncology services and to new and accepted drugs, implementation of already existing workforce plans for the oncology workforce and for radiation oncology, revision of the system of support for the travel of patients and carers to receive care, and special attention to equity of access, especially for indigenous Aboriginal people. The recommendations were intended for consideration by the Commonwealth Minister for Health and Ageing in concert with state and territory health authorities.

### Strengthening Cancer Care

“Strengthening Cancer Care” was released prior to the federal election in 2004 by the former Coalition government [76]. This document outlined a series of initiatives to improve cancer care in Australia, including supporting Australians living with cancer and the professionals who care for them, enhancing screening and prevention efforts in bowel and skin cancer, prevention of smoking in pregnancy, better access to Pap smears for cervical cancer, ensuring better coordination for national cancer efforts, and more research funding dedicated to cancer and cancer care. Funding of almost $190 million for these cancer care initiatives in the five years to 2008-2009 had been approved. State cancer plans now exist for each mainland state in Australia. The Northern Territory has recently commissioned a group to develop its cancer plan. These plans cover the spectrum of cancer control activity from prevention and screening to palliation and rehabilitation. They provide a framework for benchmarking services and for prioritising funding initiatives.

## FUTURE VISION AND DIRECTIONS OF CANCER CARE IN AUSTRALIA

Cancer outcomes in Australia are among the best in the world but there remains significant morbidity, mortality and expenses involved. The major issues for Australia are quality of service, distribution and access to services and the increasing cost of drugs and technology. Strategies to improve the quality of service include accreditation of training institutions, credentialing of practitioners and improvements in information technology. Accreditation and credentialing recognise the contribution of expertise to better outcomes. The current debate surrounds the best method to enforce accreditation and the content of the standards that will be used, such as that outlined and suggested in the ‘Cancer Services Framework for Victoria’ report [72]. Meaningful standards will also require significantly better information management. Currently only incidence, mortality and survival data are routinely collected and reported. Staging and treatment data are only available from a limited number of hospital-based registries such as those in South Australia. There are also initiatives to develop and assess cancer multi-disciplinary teams with more objective criteria and performance benchmarks [77].

Australia has an extremely dispersed geographic population. One-third of the population lives outside major metropolitan centres. Recent initiatives have sought to improve the provision of services in rural and regional areas with the opening of new cancer centres in many major country towns in Victoria and NSW. There is no radiotherapy service in Darwin, the capital of the Northern Territory, and cancer patients have to travel a minimum of four hours (by flight) to the nearest service. Providing services in isolated centres means it is more expensive because a minimum of two linear accelerators are required to prevent interruptions to treatment by equipment breakdowns. Staffing may be difficult because of the overall shortages of skilled staff, particularly at a senior level. Establishment of cancer networks, linking metropolitan areas with more regional and remote areas, in terms of staff, training, and sharing of other resources facilitated by appropriate information and communication technologies (ICT) may be one of the potential solutions to the challenges burgeoning cancer care in an ageing population. The high cost of new biological agents, as well as new imaging and treatment technology presents a major challenge to health services that are already short of funding. Technological innovations such as IMRT may prolong treatment duration and reduce patient throughput and thus decrease treatment capacity. Technological innovation is often introduced without evidence of benefit and safety that would be required for new drugs.

In summary, cancer care in Australia, as measured by selected outcomes, are among the best in the world; however there continues to be evidence of health inequalities especially among patients residing in regional and remote areas, the indigenous population and those of lower socio-economic classes. Ongoing implementation of the many national and state cancer control plans and evaluation of their effectiveness will be needed to pursue the goal of optimal cancer care.
